# Supplementary material for: Deconstructing allostery by computational assessment of the binding determinants of allosteric PTP1B modulators
Source: Commun Chem. 2023 Jun 15;6:125. doi: 10.1038/s42004-023-00926-1 (PMC10272186; doi:10.1038/s42004-023-00926-1)
Supplement: Supplementary file 2 — Supplementary Information [file 42004_2023_926_MOESM2_ESM.pdf]

Deconstructing allostery by computational assessment of the binding determinants of allosteric PTP1B modulators

Adele Hardie[a], Benjamin P. Cossins *[b], Silvia Lovera[c], and Julien Michel[a,†]

[a]EaStChem School of Chemistry, Joseph Black Building, University of Edinburgh, Edinburgh, EH9 3FJ, UK
[b]UCB Pharma, 216 Bath Road, Slough, UK
[c]UCB Pharma, Chemin du Foriest 1, 1420 Braine-l'Alleud, Belgium
[†]email: julien.michel@ed.ac.uk

*present address: Exscientia, The Schrödinger Building, Oxford Science Park, Oxford, UK

System	peptide	ligand PDB ID	ligand charge
Apo	None	None	None
Reference	Yes	None	None
1	Yes	1T4J	-1
2	Yes	1T48	-1
3	Yes	6B95	0
4	Yes	5QDL	0

Supplementary Table 1: Structure information for each system

Ligand	Restraint atoms	Restraint bounds/Å	Force constants/kcal mol^{-1}
2	2(N01) and Glu276(Cδ)	2.5, 3.0, 4.0, 4.5	0, 300
2	2(O19) and Asn193(Cγ)	2.5, 3.0, 4.0, 4.5	0, 300
3r	3(S19) and Cys197(S)	2.5, 3.0, 4.0, 4.5	0, 50
4	4(N18) and Cys197(S)	2.5, 3.0, 4.0, 4.5	0, 50

Supplementary Table 2: Flat bottom restraint parameters

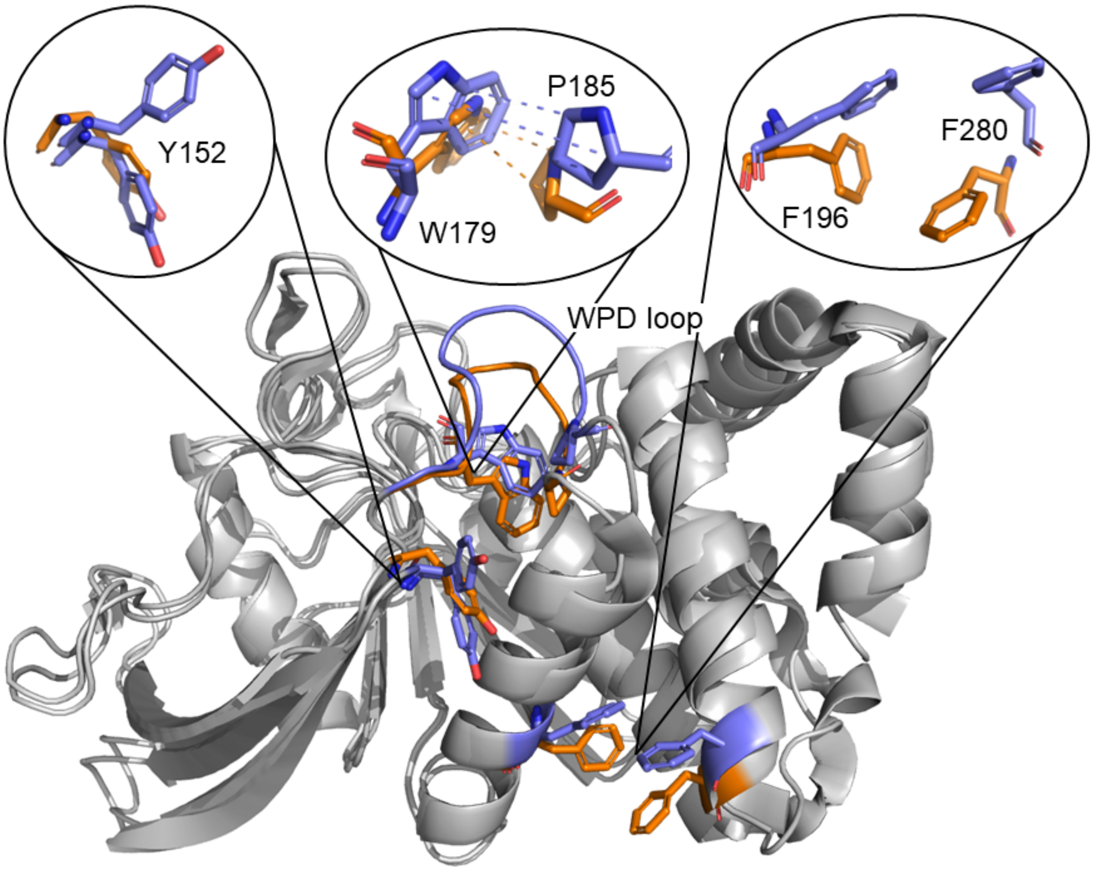

Supplementary Figure 1: Steered molecular dynamics (sMD) collective variables (CVs): WPD loop heavy atom RMSD, Y152 $\chi 1$ angle, P185 stacking to W179 (defined as the absolute difference between the P185(Cδ)-W179(Cϵ) and P185(Cα)-W179(Cδ1) distances), and F196 stacking to F280 (defined as the F196(Cγ)-F280(Cγ) distance).

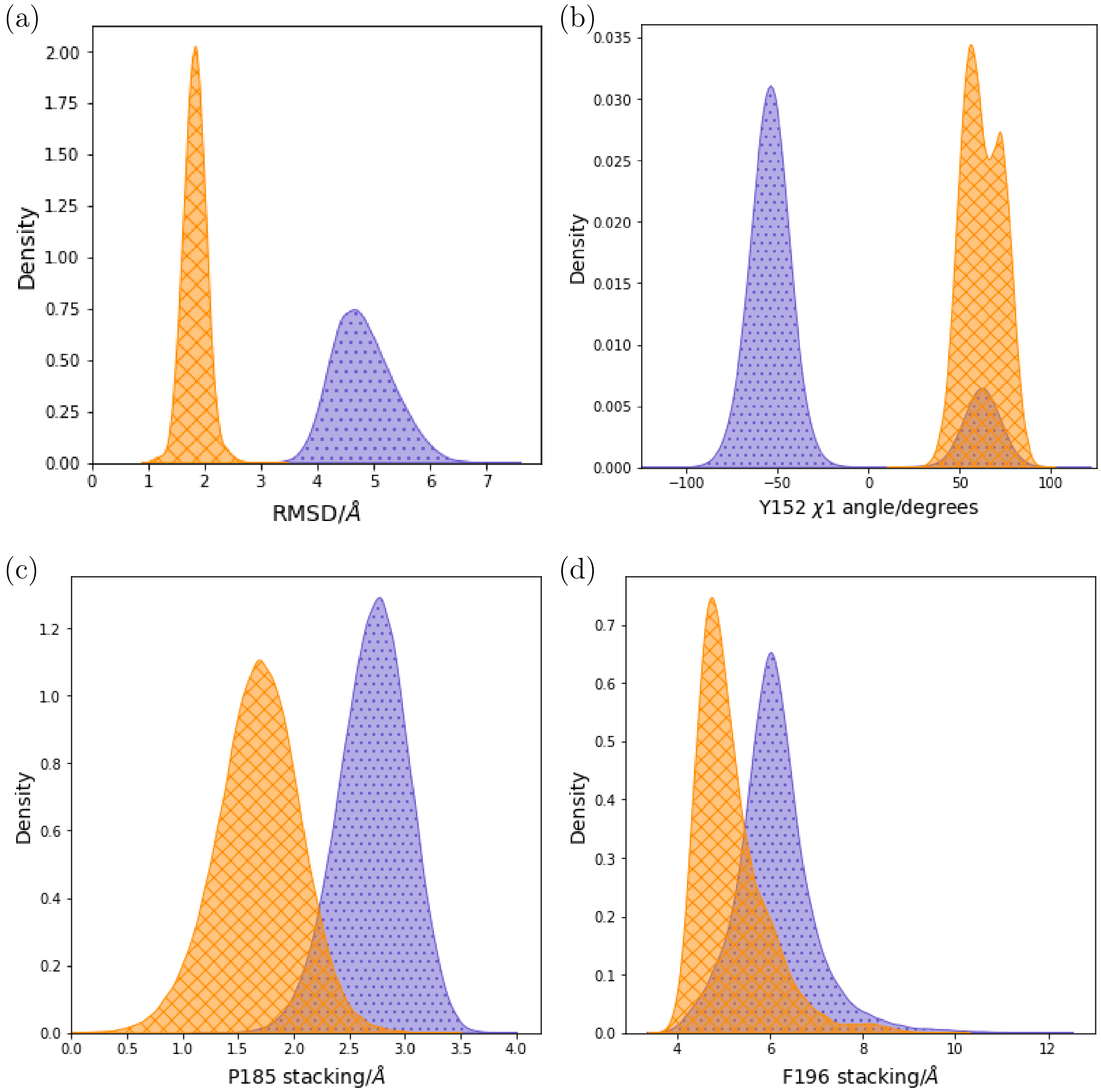

Supplementary Figure 2: The collective variables outlined in 1 during 1 μs equilibrium MD simulations of PTP1B with the peptide substrate when the WPD loop was open (blue, dots) and closed (orange, crosses). **a** WPD loop heavy atom RMSD to PTP1B with the closed loop conformation. **b** Y152 *chi*1 angle. **c** P185 stacking to W179 distance, which is defined as the absolute difference between the P185(Cδ)-W179(Cϵ) and P185(Cα)-W179(Cδ1) distances. **d** F196 stacking to F280 stacking, which is defined as the F196(Cγ)-F280(Cγ) distance.

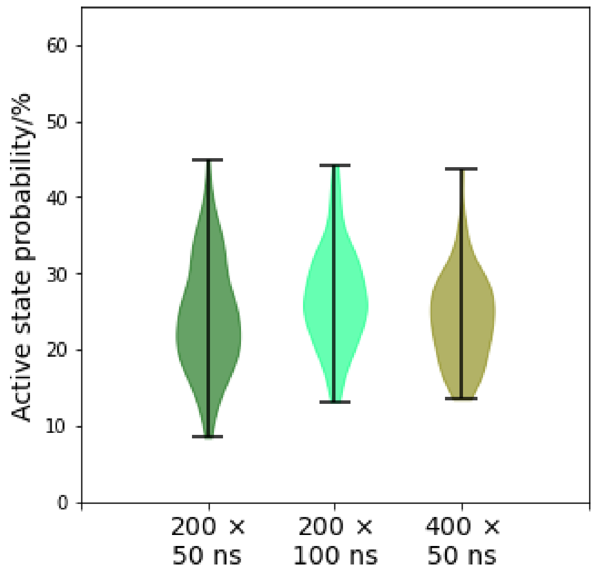

Supplementary Figure 3: MSM of PTP1B with compound 2r when seeded MD duration was increased from 50 ns to 100 ns (20 μ total sampling time), and the number of seeded MD trajectories was increased from 200 to 400 (20 μ total sampling time), compared to original sampling.

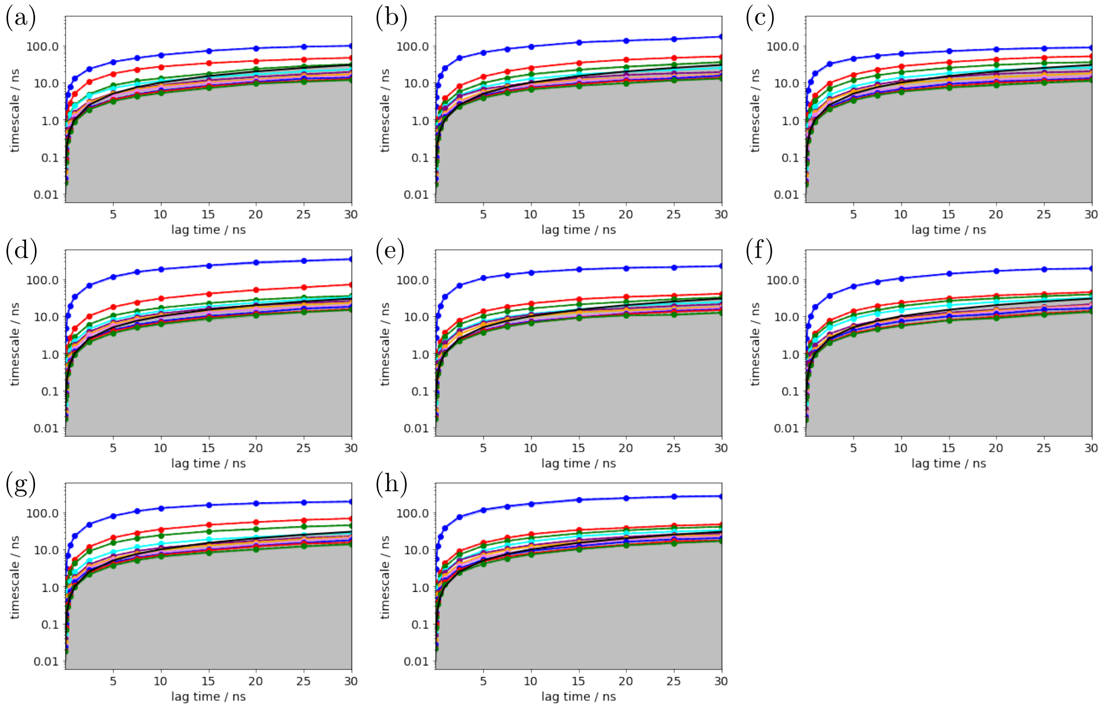

Supplementary Figure 4: Implied timescales (ITS) of each MSM: **(a)** apo **(b)** reference **(c)** 1 **(d)** 2 **(e)** 2r **(f)** 3 **(g)** 3u **(h)** 4

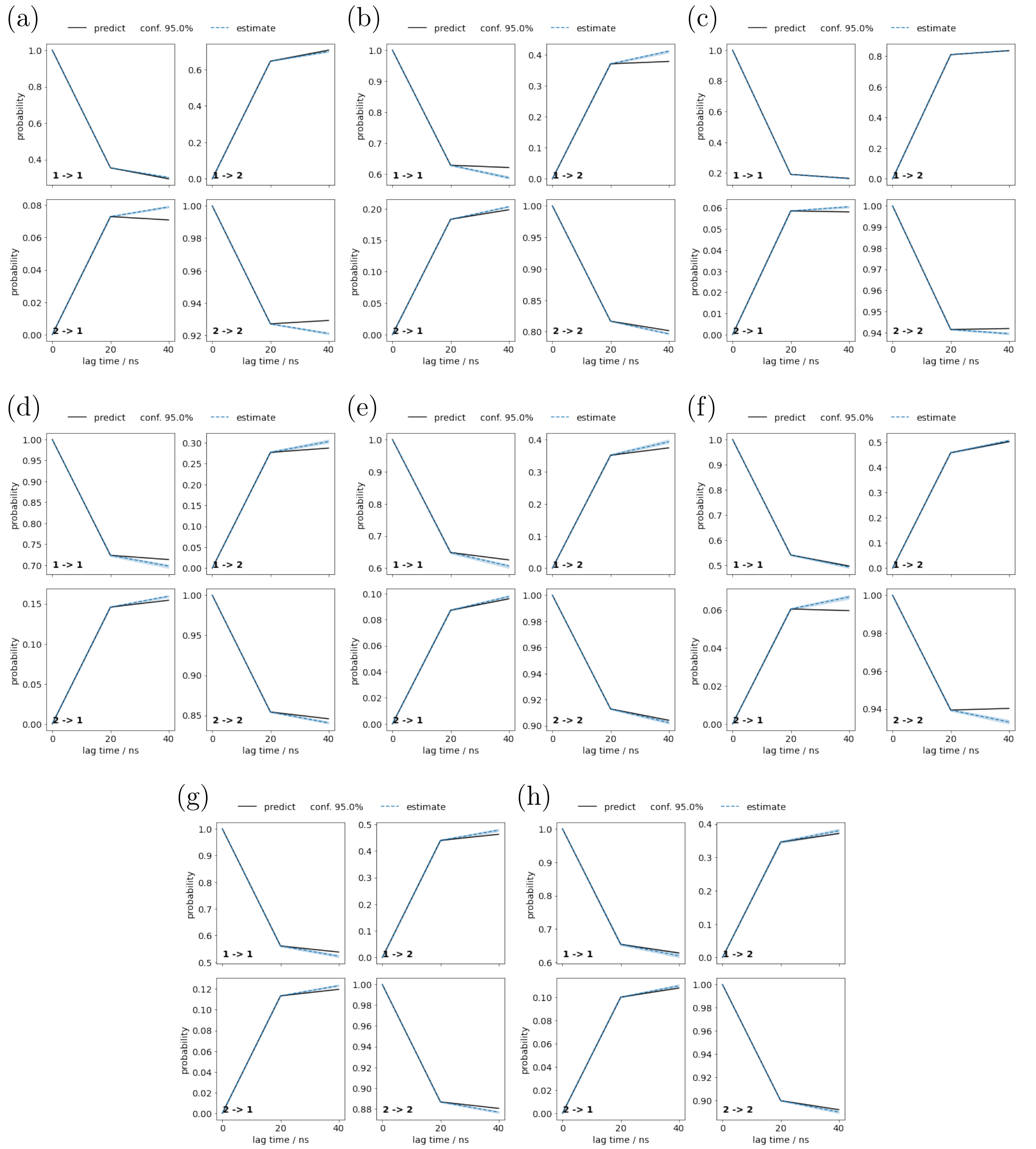

Supplementary Figure 5: Chapman-Kolmogorov test of each MSM: **(a)** apo **(b)** reference **(c)** 1 **(d)** 2 **(e)** 2r **(f)** 3 **(g)** 3u **(h)** 4

(a)

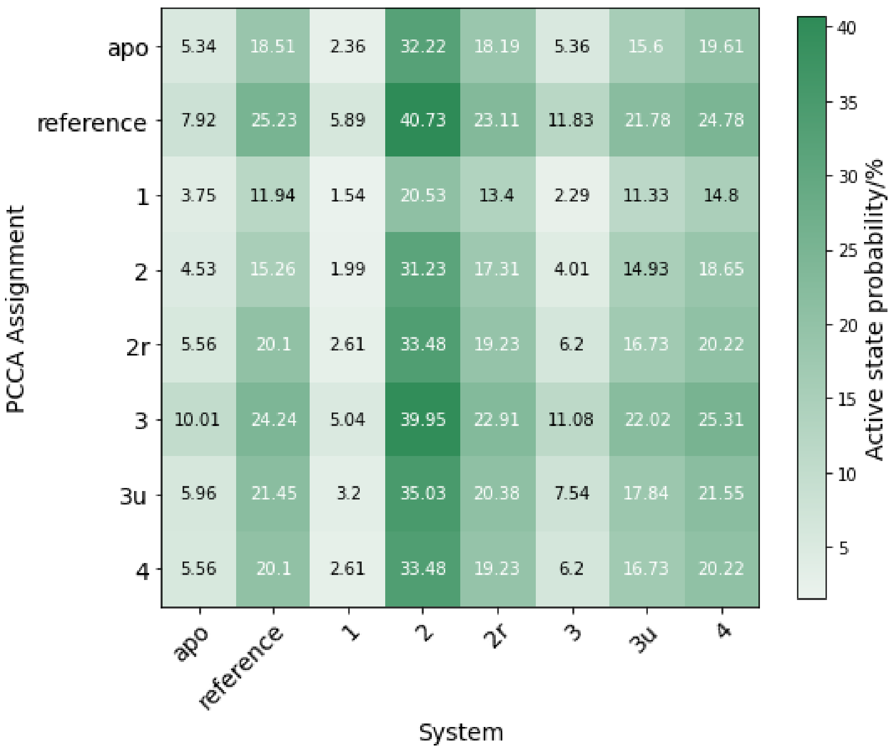

(b)

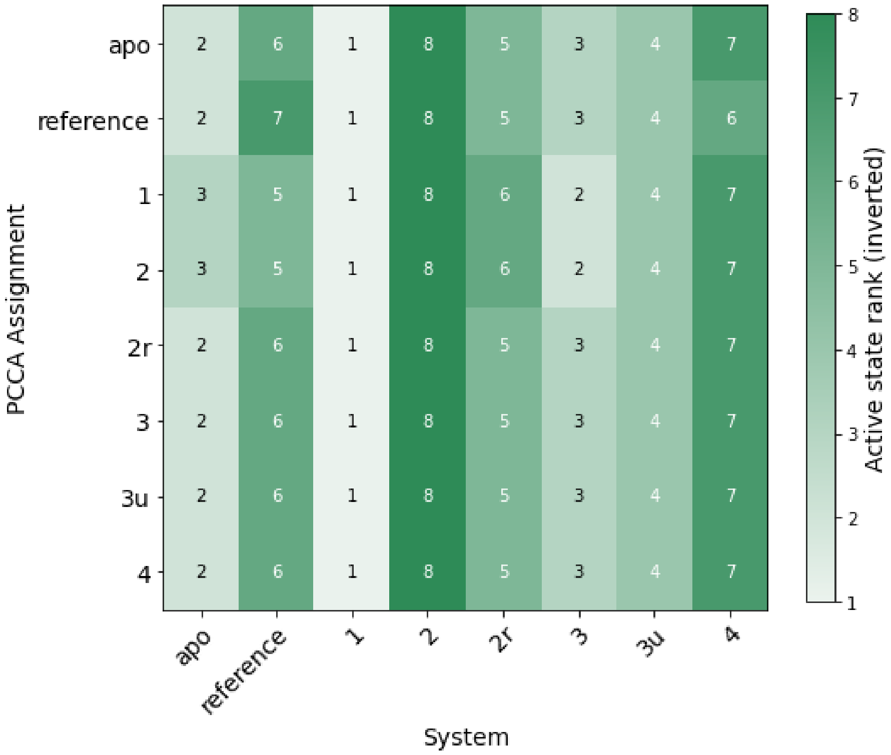

Supplementary Figure 6: Active state probabilities **(a)** and inverse active state ranking (1 - lowest, 7 - highest) **(b)** when using PCCA assignments from each MSM to assign the active state.

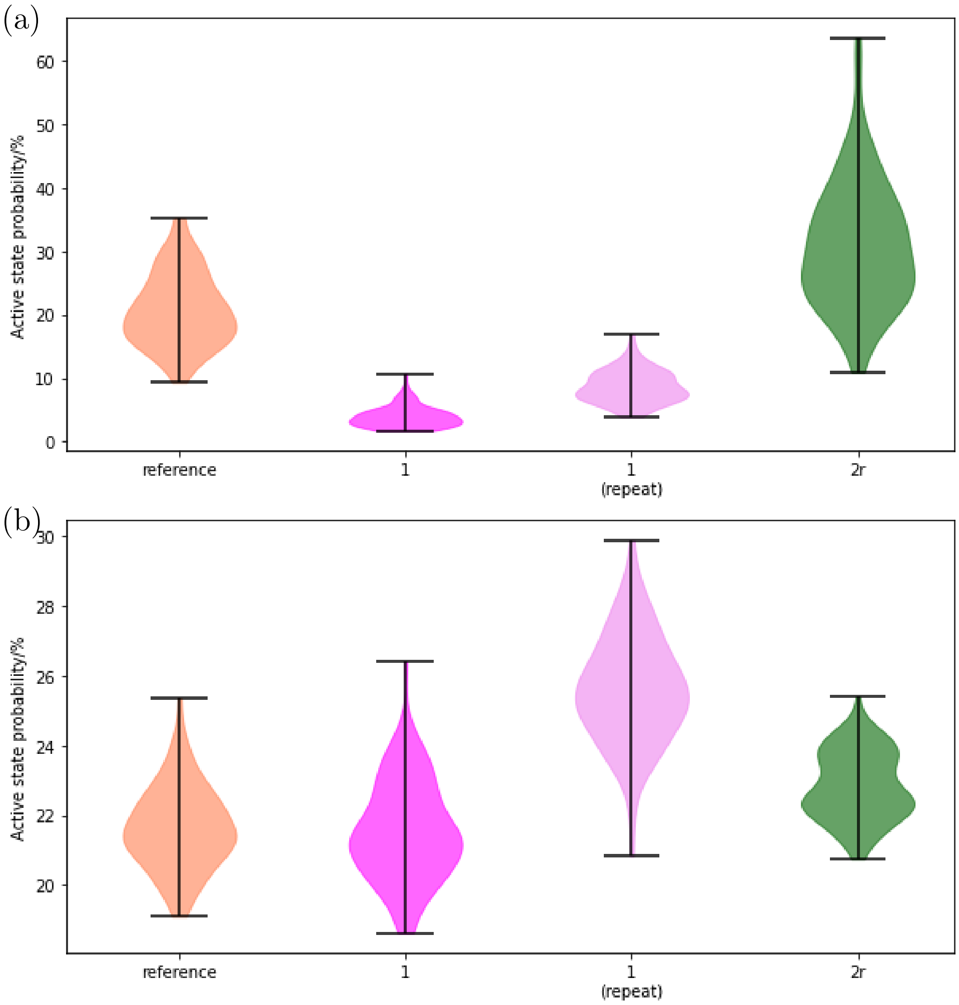

Supplementary Figure 7: Comparison of MSM results when different CVs shown in Figure 1 were used for sMD. **(a)** All 4 CVs **(b)** WPD loop only. When only the WPD loop is used, the rest of the system (including the allosteric network) does not adjust to the steered change in the WPD loop conformation. Here reference, 1, a repeat of 1 and 2r systems are used as examples. When all 4 CVs are used, the results for compound 1 are reproducible, and it is correctly modelled as an inhibitor.
